# Robotic-Assisted Total Knee Arthroplasty in Complex Primary and Revision Cases: A Systematic Review

**DOI:** 10.1007/s12178-026-10026-x

**Published:** 2026-04-09

**Authors:** Emmanuel Olaonipekun, Kourosh Movahedi, Prushoth Vivekanantha, Paul Kooner, Seper Ekhtiari

**Affiliations:** 1 School of Medicine, Royal College of Surgeons in Ireland, 123 St Stephen’s Green, Dublin 2, Dublin, D02 YN77 Ireland; 2 School of Medicine, American University of the Caribbean, Cupecoy, Sint Maarten Netherlands; 3 Division of Orthopaedic Surgery, Department of Surgery, McMaster University, Hamilton, ON Canada; 4 Granovsky Gluskin Division of Orthopaedics, Department of Surgery, Mount Sinai Hospital, Sinai Health System, Toronto, Canada; 5 Division of Orthopaedic Surgery, Department of Surgery, Temerty Faculty of Medicine, University of Toronto, Toronto, Canada

**Keywords:** Robotic-assisted TKA, Complex knee arthroplasty, Revision TKA, Varus deformity, Valgus deformity, Obesity, Patient-reported outcomes

## Abstract

**Purpose of Review:**

This study systematically reviews outcomes of robotic-assisted total knee arthroplasty (rTKA) in complex primary and revision cases, compared with conventional TKA (cTKA). Complex cases include severe coronal deformity, high BMI, fixed flexion deformity, and revision arthroplasty. Outcomes assessed included coronal alignment, perioperative metrics, and patient-reported outcomes.

**Recent Findings:**

Nineteen studies comprising 2,482 patients (2,535 knees: 1,931 rTKA, 604 cTKA) were included. Robotic-assisted TKA consistently restored coronal alignment, with greatest improvements observed in moderate-to-severe varus and valgus deformities. In revision cases, robotic assistance achieved near-neutral hip–knee–ankle alignment (mean deviation − 1.05°), low complication rates (4–17.9%), and high implant survival (97.1%). In obese patients, robotic-assisted TKA improved alignment precision, though functional outcomes were similar to conventional techniques. Robotic systems reduced the need for extensive soft tissue releases and enabled reliable early functional recovery. Most studies were retrospective, with moderate-to-serious risk of bias due to confounding and heterogeneity in patient populations, surgical complexity, and robotic platforms.

**Summary:**

Robotic-assisted TKA reliably restored coronal alignment and achieved perioperative andpatient-reported outcomes comparable to conventional techniques in complex primary andrevision cases. Alignment advantages were particularly evident in severe deformities oranatomically challenging knees. While these findings are encouraging, they should be3interpreted in the context of predominantly retrospective data, heterogeneous outcomereporting, and limited long-term follow-up. Future prospective, longitudinal randomizedstudies with standardized reporting are needed to confirm the impact of robotic-assistedapproaches on long-term functional outcomes and implant survival, in complex TKA.

**Supplementary Information:**

The online version contains supplementary material available at 10.1007/s12178-026-10026-x.

## Introduction

Over the past decade, the use of robotic assisted total knee arthroplasty (rTKA) has increased globally [1]. Recent registry data shows trends of increased use of rTKA ranging from 5 to 35% of all primary knee replacement surgeries across Canada, Australia, and the United States (USA) [2–4]. This technology is intended to improve precision of surgical tasks such as implant positioning, performing bone cuts and evaluating final range of motion and joint balance. However, it is unclear in the literature if this leads to superior clinical outcomes after TKA.

Despite the proven effectiveness of conventional TKA (cTKA), a considerable number of patients are still dissatisfied after surgery (10–15%) [5]. Patient and surgical factors such as limb alignment, degree of deformity, postoperative implant position and soft tissue balancing are known to influence patient satisfaction and outcomes [5, 6]. The enabling technology of rTKA allows for real time intraoperative balance assessment and subsequent component positioning that is personalized to patient specific ligamentous behaviour. However, long term randomized controlled trials (RCTs) are unable to demonstrate a clear superiority in patient reported outcome scores [7, 8].

In addition to improving precision in routine primary TKA, rTKA may also have applications in complex and revision TKA. The ability to bypass intramedullary instrumentation, and additional feedback including overall limb alignment and hip centre can contribute to surgical workflow in these scenarios. Some modern rTKA platforms now offer specific features to enable complex primary and revision TKA. There is a paucity of literature examining the role of rTKA in complex cases. Patients with a high body mass index (BMI), severe valgus or varus deformity, or revision cases with previously retained hardware have been shown to have inferior outcomes when compared to the routine primary TKA [5, 9–11]. These patients often experience increased perioperative complications including increased hospital length of stay, blood loss and operative times [10, 12]. Furthermore, there is an increased incidence of septic and aseptic loosening and decreased postoperative outcome scores within this population. It is unclear how the use of enabling technology of robotic assisted TKA may influence the outcomes within this subset patient population.

The purpose of this systematic review is to evaluate the outcomes of rTKA in complex primary and revision total knee arthroplasty when compared to conventional techniques. These cases present unique and challenging surgical complexity that may benefit from robotic assistance. We aim to outline the role and outcomes of robotic assisted TKA in restoring knee kinematics pre and postoperatively, perioperative complications and overall patient reported outcome measures.

## Methods

### Search Strategy

This systematic review was conducted according to the Cochrane Handbook for Systematic Reviews of Interventions and reported within the guidelines of the Preferred Reporting Items for Systematic Reviews and Meta-Analyses (PRIMSA) [13, 14]. The study protocol was prospectively registered on the PROSPERO international prospective register of systematic reviews (registration number: CRD420251274473).

Study selection was carried out by two authors (EO and KM) who searched Medline, Central and Embase databases to identify articles for review. The search was performed on 15/01/2026. The search strategy combined terms related to robotic-assisted surgery, total knee arthroplasty, and complex knee arthroplasty populations. Keywords included robotic surgery terminology (such as robotic, robot-assisted, MAKO, ROSA, VELYS, NAVIO, and CORI), combined with terms related to total knee arthroplasty (including total knee arthroplasty and TKA), as well as terms describing complex cases such as complex, severe deformity, varus, valgus, obesity, post-traumatic, prior surgery, osteotomy, and revision. The full search strategy for each database is provided in (Supplementary Table 1). Title and abstract screening as well as full-text review were performed independently by two reviewers (EO and KM), with disagreements at the title and abstract stage resolved by automatic inclusion, while disagreements at the full text stage were resolved through discussion and consensus with a third, more senior author (PK).

Complex TKA was defined as TKA for the following indications: severe coronal deformity (varus or valgus > 10–15º), high BMI > 35 kg/m^2^, fixed flexion deformity, post-traumatic arthritis with prior knee procedure (previous osteotomy or retained hardware after fracture), or revision TKA.

Studies were eligible for inclusion if they met the following criteria: original clinical studies reporting on robotic-assisted primary TKA in adult patients (≥ 18 years) undergoing complex TKA as defined above; randomized controlled trials, prospective cohort studies, or retrospective cohort studies; and studies reporting clinical or radiographic outcomes including hip–knee–ankle alignment (HKA), patient-reported outcome measures (such as Knee Society Score [KSS], Western Ontario and McMaster Universities Osteoarthritis Index [WOMAC], or Knee Injury and Osteoarthritis Outcome Score [KOOS]), perioperative outcomes (including operative time, blood loss, and length of hospital stay), or complications. Only studies published from 2020 to the date of search execution were considered. Studies were excluded if they were cadaveric or biomechanical investigations, case reports or small case series including fewer than 10 patients, or studies that did not evaluate complex TKA according to the predefined criteria.

### Verification of Title and Abstract Screening with Artificial Intelligence

Records retrieved from all four databases were combined and de-duplicated using a custom Python script. Duplicate citations were identified using a sequential rule-based approach, first by exact DOI matching, followed by matching of title and year of publication if DOI was not available. The script generated a final deduplicated record set with logs of removed duplicate citations and summary of records retained and excluded at each step.

De-duplicated records then underwent artificial intelligence-assisted title and abstract verification using two large language models (LLMs), GPT-5.2 (OpenAI) and Claude 4.5 Opus (Anthropic, San Francisco, CA, USA). Each model was prompted independently using the same inclusion and exclusion criteria provided to human reviewers for robot-assisted TKA in complex or revision populations and returned binary decisions on inclusion versus exclusion. The AI outputs were generated in parallel with human reviewers and were used as supplementary verification. The workflow was adapted from a prior proof-of-concept work evaluating LLMs for automated title and abstract screening in Orthopaedic systematic reviews using GPT-5 [15]. The prompt for the models can be found in Appendix Table 1.

### Risk of Bias Assessment

Risk of bias was assessed using the ROBINS-I for non-randomized studies and the Cochrane Risk of Bias 2 (RoB 2) for randomized control trials. Two authors independently evaluated each study across the relevant bias domains. For non-randomized studies, the ROBINS-I tool assessed bias related to confounding, participant selection, intervention classification, deviations from intended interventions, missing data, outcome measurement, and selective reporting. The single randomized controlled trial was assessed using the RoB 2 framework, which evaluates bias arising from the randomization process, allocation concealment, selective reporting, incomplete outcome data, blinding and outcome assessment. Any discrepancies between reviewers were resolved through discussion, with final consensus reached in consultation with the senior author.

### Outcome measures

The primary objectives were to report the clinical and radiographic outcomes of robotic versus cTKA in complex primary or revision cases. Outcome measures collected included accuracy of component positioning, restoration of pre and postoperative alignment, functional outcome scores and complications. Secondary objectives included reporting demographic data, robotic platforms utilized, and qualitative assessment of studies reported.

### Statistical Analysis

Due to heterogeneity in study design, patient populations, and outcome reporting across the included studies, a formal meta-analysis was not performed. Outcomes were therefore synthesized using a descriptive approach. Continuous variables reported across studies were summarized using reported means and standard deviations where available, while categorical outcomes were summarized using proportions. Where appropriate, simple averages were calculated across studies reporting comparable outcomes. Variability in outcome definitions and reporting across domains, including radiographic alignment, patient-reported outcomes, and complications, limited direct quantitative comparisons between studies.

## Results

### Study Selection

**Fig. 1:**
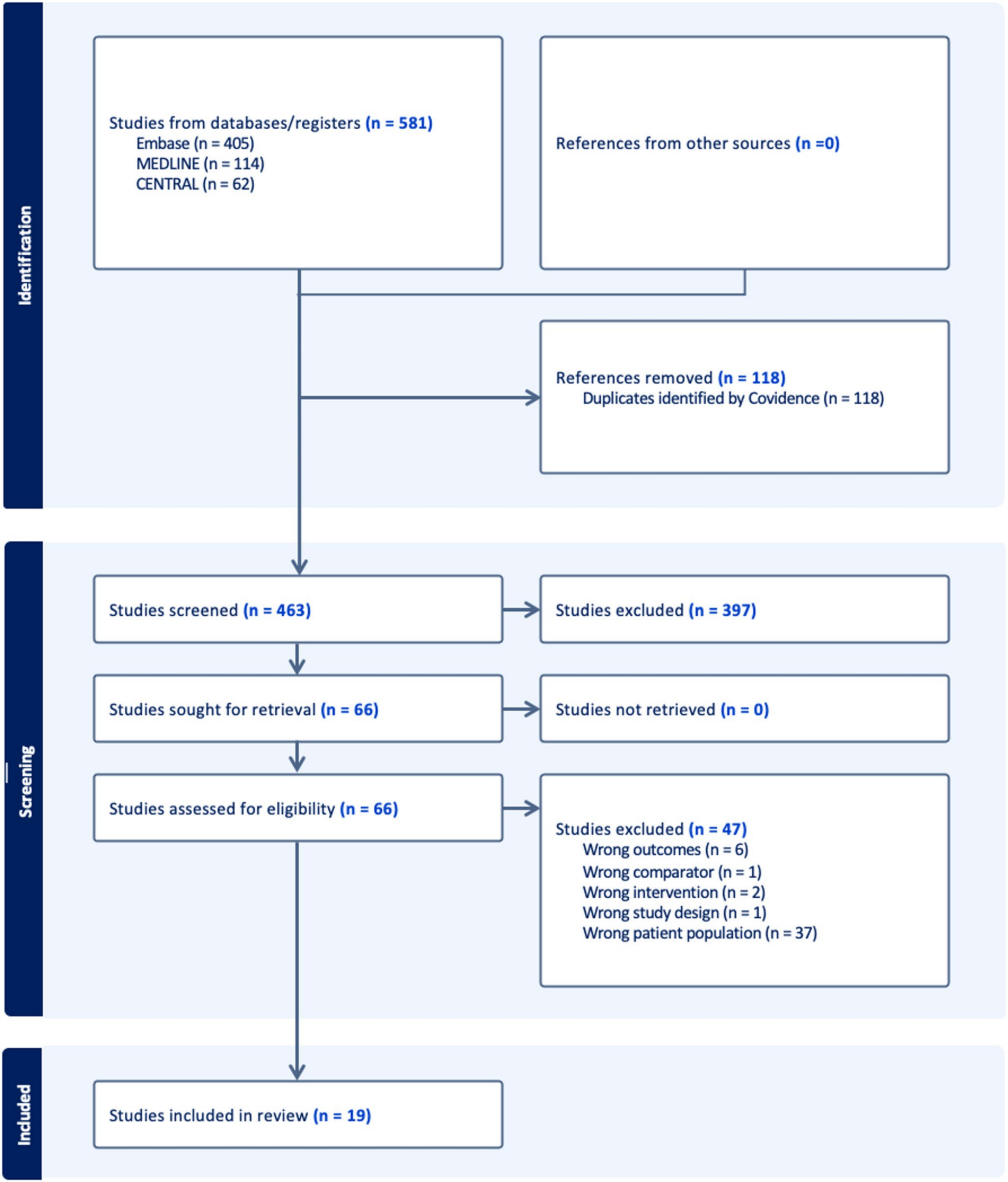
PRISMA Flow Diagram of Study Selection

The study selection process followed the Preferred Reporting Items for Systematic Reviews and Meta-Analyses (PRISMA) guidelines and is summarized in Fig. 1. A total of 581 records were identified through database searches. After removal of 118 duplicate records, 458 studies remained for title and abstract screening. Following screening, 66 studies were retrieved for full-text assessment. Of these, 47 studies were excluded, leading to 19 studies that met the inclusion criteria and were included in the final systematic review.

### Verification of Title and Abstract Screening with Artificial Intelligence

Following de-duplication, 406 unique citations for title and abstract screening remained and were passed through the artificial intelligence screeners. GPT-5.2 and Claude 4.5 Opus both included 17 studies, with 15 being part of the 19 final studies included by the humans. The two other studies did not meet the inclusion criteria and did not pass through to final inclusion. GPT included one study that Claude excluded while GPT excluded four studies that Claude included. Amongst these five, one study was part of the 19 final included studies by humans. Three studies of the final 19 were missed by both LLMs, including Vecham et al., Marchand et al., and Gregori et al. [16–18].

### Study Characteristics

A total of 19 studies met the inclusion criteria and were included in the final analysis [16–34]. These studies comprised 2,482 patients (2,535 knees) including 1,931 robotic-assisted total knee arthroplasties and 604 conventional total knee arthroplasties. The characteristics of the included studies are summarized in Table 1. Most included studies were retrospective cohort studies (*n* = 12), followed by prospective cohort studies (*n* = 4), case–control studies (*n* = 2), and one RCT. The studies were published between 2021 and 2026, with an increasing number of publications in recent years reflecting the growing adoption of robotic technology in complex knee arthroplasty. Geographically, the majority of studies originated from the United States (*n* = 8), followed by China (*n* = 4). Additional studies were conducted in Italy (*n* = 2), France (*n* = 2), the United Kingdom (*n* = 1), Israel (*n* = 1), and India (*n* = 1).

**Table 1:** Baseline Patient Characteristics of Included Studies

Study	Number of Patients	Mean Age (years)	Male *n* (%)	Mean BMI (kg/m²)	Pre-op HKA (°) ± SD
Andriollo 2025	35	69.7 ± 7.0	10 (24.2)	25.5 ± 5.3	NR
Cabell 2026	84	67.5 ± 9.6	33 (39.3)	32.6 ± 5.1	-2.6 ± 5.9
Chang 2026	66	67.7 ± 9.3	18 (27.2)	31.5	NR
Chapron 2025	81	72.2 ± 7.8	18 (24.3)	27.8 ± 4.2	NR
Chen 2023	106	67.6 ± 6.8	21 (19.8)	26.8 ± 3.7	7.6 ± 3.9
Cochrane 2024	115	65	50 (43)	32.4	NR
Fang 2024	194	65.3	70 (28.6)	NR	11.9 ± NR
Feng 2025	218	65 ± 6.0	44 (20.5)	26.8 ± 2.9	NR
Gorur 2024	73	69 ± 7.5	7 (9.4)	29.9 ± 6.1	NR
Gregori 2025	58	70 (IQR 64–74.5)	19 (33)	26.6 ± 5.6	187 ± NR
Marchand 2021	152	NR	NR	NR	NR
McCormick 2023	350	66.3 ± 8.9	144 (41.1)	31.8 ± 5.9	NR
Pacchiarotti 2025	120	71.5 ± 5.4	71 (59)	27.5 ± 3.5	176.58 ± 6.83*
Richardson 2024	119	63.5 ± 7.97	29 (24.3)	39.4 ± 3.6	8.89 ± 5.06
Schiman 2024	123	69 ± 9.6	41 (33.3)	30.6 ± 5.7	6.7 ± 6.5
Tian 2025	123	NR	NR	NR	slight: 1.56 ± 0.95; severe: 2.09 ± 1.18
Tuecking 2021	20	NR	NR	NR	NR
Winniger 2023	NR	NR	NR	NR	NR

Values are reported as mean ± standard deviation unless otherwise stated. *BMI* Body mass index (kg/m²), *HKA* Hip–knee–ankle angle (°), representing coronal alignment of the lower limb, *IQR* Interquartile range, *NR* Not reported

Follow-up was reported in 11 of 19 included studies, with durations ranging from 3 to 47.2 months. Across the included studies, the mean patient age was 67.3 years (SD 7.5), and the mean body mass index (BMI) was 29.8 kg/m² (SD 6.1). The overall proportion of male patients was 31.3%. Baseline demographic characteristics across studies are summarized in Table 2. The included studies evaluated rTKA in a range of complex arthroplasty scenarios, including severe varus and valgus deformities, obesity, fixed flexion deformity, and revision total knee arthroplasty. The robotic platforms used varied across studies and included commonly utilized systems such as the MAKO system by Stryker (Kalamazoo, Michigan USA), the NAVIO/CORI from Smith & Nephew (Andover, Massachusetts USA) or ROSA from Zimmer Biomet (Warsaw, Indiana USA), as well as other region-specific robotic platforms.

**Table 2:** Characteristics of Included Studies

Study	Country	Study Design	Level of Evidence	Setting	Total Sample	Robotic Knees	Conventional Knees	Robotic Platform	Population / Complexity	Follow-up (months)
Andriollo 2025	Italy	Prospective Cohort	II	Single center	35	35	N/A	ROSA (Zimmer Biomet)	Revision UKA → TKA	31.3 ± 12.1
Cabell 2026	USA	Retrospective Cohort	III	Single center	84	84	N/A	CORI	Revision TKA	12 (SD NR)
Chang 2026	USA	Case-Control	III	Single center	66	24	42	MAKO	Revision TKA	47.2 ± 24.3
Chapron 2025	France	Prospective Cohort	IV	Single center	81	81	NR	MAKO	Severe valgus deformity	NR
Chen 2023	China	Retrospective Cohort	III	Single center	106	106	NR	MAKO	Varus deformity	NR
Cochrane 2024	USA	Retrospective Cohort	III	Multicenter	115	115	NR	CORI	Revision TKA	13 (SD NR)
Fang 2024	China	Retrospective Cohort	III	Single center	194*	244*	NR	MAKO	Severe varus deformity	NR
Feng 2025	China	Retrospective Cohort	III	Single center	218	84	134	TINAVI Robot	Severe varus deformity	3 (SD NR)
Gorur 2024	USA	Retrospective Cohort	NR	Single center	73	44	30	CORI / NAVIO	Valgus deformity (> 5°)	12 (SD NR)
Gregori 2025	France	Retrospective Cohort	NR	Single center	58	58	NR	MAKO	Valgus deformity	Median 18 (IQR 13.75–27)
Marchand 2021	USA	Prospective Cohort	IV	Single center	152	152	NR	MAKO	Valgus deformity	12 (SD NR)
McCormick 2023	USA	Retrospective Cohort	III	Single center	350	186	164	MAKO	Obese / morbidly obese	6–24
Pacchiarotti 2025	Italy	Retrospective Cohort	III	Single center	120	120	NR	MAKO	Fixed flexion deformity	NR
Richardson 2024	USA	Retrospective Cohort	NR	Single center	119	60	59	MAKO	Obese patients	NR
Schiman 2024	Israel	Retrospective Cohort	III	Single center	123	123	NR	Imageless robotic system	Varus deformity	NR
Tian 2023	China	Randomized Controlled Trial	I	Multicenter	144	72	72	Hangzhou Jianjia Robot	Severe deformity	3 (SD NR)
Tuecking 2021	UK	Case-Control	III	Single center	40	40	NR	NAVIO / Journey II	Revision UKA → TKA	NR
Vecham 2025	India	Prospective Cohort	II	Single center	200	100	100	MAKO vs. Meril CVUS	Severe varus deformity	NR
Winniger 2023	USA	Retrospective Cohort	III	Single center	206	103	103	ROSA	Varus deformity	3–6

*NR* Not reported, *IQR* Interquartile range, *TKA* Total knee arthroplasty, *UKA* Unicompartmental knee arthroplasty. Follow-up duration reported in months unless otherwise stated. Study design, level of evidence, and robotic platforms are reported as described in the original publications

### Outcomes in Revision Knee Arthroplasty

Five studies including 340 revision knees (298 robotic assisted, 42 conventional) evaluated the use of robotic-assisted arthroplasty in revision knee surgery [19–23]. Indications for revision surgery were failed primary TKA from aseptic and septic causes [19, 20, 23] or OA progression and component degradation in the context of previous (UKA) [21, 22]. The studies primarily aimed to assess the feasibility and effectiveness of robotic-assisted techniques in achieving accurate radiographic alignment, restoring joint line, optimizing perioperative outcomes, and improving patient-reported outcomes [19, 21, 22].

Postoperative alignment was reported in two studies. After converting HKA measurements to deviation from neutral alignment (− 1.4° ± 3.2° (SD), − 0.7 ± 2.3(SD)) [19, 22], the pooled mean postoperative HKA was − 1.05°, indicating overall restoration close to neutral mechanical alignment (Table 3). Mean MPTA was reported in two studies: 89.8° ± 1.8° (SD) and 88.5° ± 1.5° (SD), with an overall mean of 89.15° (Table 3) [19, 21]. Overall, radiographic restoration of coronal alignment appeared accurate across cohorts, with alignment close to neutral mechanical axis.

**Table 3:** Clinical and Radiographic Outcomes Following Robotic-Assisted Revision TKA

Study	*N* (Robotic)	Post-op HKA (°)	Deviation from Neutral (°)	Post-op MPTA (°)	Change in KOOS Jr	OKS	Operative Time (min)	LOS (days)	Complication Rate (%)	Re-revision Rate (%)
Cabell 2026	84	178.6 ± 3.2*	−1.4 ± 3.2	89.8 ± 1.8	−4.0 ± 5.3†	NR	NR	2.3 ± 2.5	17.9	NR
Cochrane 2024	115	NR	NR	NR	NR	NR	186	2	7	3
Tuecking 2021	20	NR	NR	88.5 ± 1.5	NR	NR	76 ± 11.2	NR	NR	NR
Andriollo 2025	35	179.3 ± 2.3	−0.7 ± 2.3	NR	NR	41.5 ± 4.3	116 ± 19.6	4.2 ± 1.7	4	NR
Chang 2026	24	NR	NR	NR	NR	NR	171.7	2.5	4.1	0
Overall / Mean	339	178.95°	−1.05°	89.15°	−4.0	41.5	137.3	2.75	8.25	1.5

*TKA* Total knee arthroplasty, *HKA* Hip–knee–ankle angle (°), coronal limb alignment, *MPTA* Medial proximal tibial angle (°), tibial component alignment, *KOOS Jr/KOOS* Knee injury and Osteoarthritis Outcome Score, *OKS* Oxford Knee Score, *LOS* Length of stay (days), *NR* Not reported. Complication (%) includes all reported postoperative complications. Overall/mean values calculated from available data

*Absolute HKA reported; deviation from neutral calculated where available

†Change in KOOS Jr represents pre- to post-operative difference; baseline mean was 15.4 ± 5.5, post-op 11.4 ± 5.3 (*p* = 0.0006)

Patient-reported outcomes were generally favourable, although reporting was variable across studies. Cabell et al. reported a mean change in KOOS Jr of − 4.0 ± 5.3, reflecting improvement in knee function postoperatively, while Andriollo et al. reported a mean OKS of 41.5 ± 4.3 (Table 3) [19, 22]. Cochrane et al. assessed PROMIS pain and depression scores, demonstrating low levels of pain and minimal depressive symptoms at final follow-up [20].

In terms of perioperative outcomes and complications, mean operative times ranged from 76 ± 11.2 min to 186 min, with an overall average of 137.3 min. Mean length of stay was 2–4.2 days, with an average of 2.75 days [20, 21]. Overall complication rates ranged from 4% to 17.9%, with implant survival reported at 97.1% at final follow up in the study by Andriollo et al. (Table 3) [22]. Re-revision rates were low, with a mean of 1.5%, and most complications were minor or managed non-operatively.

Across these five studies, robotic-assisted revision TKA demonstrated reliable restoration of coronal alignment and joint line, low complication and revision rates and favourable short-term functional outcomes [19–23]. These findings suggest that robotic assistance may support precise component placement and acceptable complication rates in complex revision cases.

### Varus Deformity

Eight studies, including 1,176 varus knees (885 robot assisted, 291 conventional), evaluated robotic-assisted TKA in patients with preoperative varus deformity [16, 24–29].These included one RCT, one prospective cohort, and six retrospective cohorts, three of which were comparative. Preoperative HKA alignment ranged from 0° to 32°. Most studies assessed postoperative alignment accuracy, coronal outliers, component positioning, soft-tissue management, and early functional outcomes. However, as outlined in Table 4, HKA angles were the most consistently reported measure and will be the main metric used to compare robotic assisted alignment correction with conventional.

**Table 4:** Preoperative vs. Postoperative HKA angles in Varus Population undergoing Total Knee Arthroplasty

Study	Total Varus Sample	*N* (Robotic)	Mean Pre-op HKA (°)	Mean Post-op HKA (°)	*N* (Convent.)	Mean Pre-op HKA (°)	Mean Post-op HKA (°)
Chen 2023	106	106	Non-Severe: 7.6, Severe: 12.6	Overall HKA deviation: 0–3° in 82.1%, 3–5° in 16%	N/A	N/A	N/A
Fang 2024	194	194	11.9 (range: 1–32)	5.1 (range: 0–19)	N/A	N/A	N/A
Feng 2025	218	84	9.5 ± 2.2	Mild: 1.4 ± 0.7, Severe: 2.2 ± 0.7	134	9.8 ± 2.8	Mild: 1.6 ± 1.0, Severe: 3.0 ± 1.4
Pacchiarotti 2025	68	41	173.97 ± 6.25	177.79 ± 2.13	27	179.19 ± 7.41	178.95 ± 2.39
Schiman 2024	123	87 (< 10°), 36 (> 10°)	Neutral: 3.6 ± 4.8, Severe: 14.5 ± 2.7	Neutral: 2.8 ± 3.3, Severe: 6.4 ± 2.7	N/A	N/A	N/A
Tian 2023	123	62	173.05 ± 6.58	Mild: 1.56 ± 0.95, Severe: 2.09 ± 1.18	61	172.03 ± 8.19	Mild: 2.25 ± 1.54, Severe: 3.57 ± 2.73
Vecham 2025	200	200	NR	NR	N/A	N/A	N/A
Wininger 2023	144	75	6.92 ± 0.84	1.95 ± 0.58	69	7.06 ± 0.98	2.75 ± 0.60

*Convent*. Conventional, *HKA* Hip–knee–ankle angle, *NR* Not reported, *N/A* Not applicable. Pre-op and post-op HKA are presented as mean ± SD or as reported by study. Subgroup classifications (mild/severe) are retained as in the original study for clarity

In terms of radiographic alignment, comparative studies demonstrated improved alignment precision with robotic assistance in patients who had greater deformity [26, 28]. Tian et al. reported significantly fewer postoperative mechanical axis outliers (> 3°) in the robotic group compared to conventional techniques (3.2% vs. 41.0%, *p* < 0.001) as well as HKA angle outliers (5.1% vs. 44.7%, *p* < 0.001) [28]. Tian et al. also demonstrated significantly improved postoperative HKA in the robotic group with severe deformity (2.09(SD 1.18) *p* < 0.001) (Table 4) compared to conventional (3.57(SD 2.73)) [28]. Similarly, Feng et al. found comparable results in mild varus cases but significantly improved coronal alignment, fewer radiographic outliers, reduced need for soft-tissue release, and superior early functional scores in severe varus knees treated robotically (*p* < 0.05) [26]. Feng et al. also measured a significant difference in postoperative MPTA in both mild and severe robotic cohorts (1.0° (SD 0.7), *p* < 0.01) vs. conventional (1.8° (SD 0.9), *p* < 0.01), and reduced HKA angle outliers in robotic cases [26]. Perioperative and patient-reported outcomes were not collected to a sufficient extent or in a consistent manner to enable further plausible comparisons within this specific deformity subgroup.

### Valgus Deformity

Six studies, including 463 valgus knees (379 robotic-assisted, 84 conventional) evaluated robotic-assisted TKA in valgus deformity. These were predominantly retrospective cohorts, with one prospective study and two case series [17, 18, 27, 30–32]. Preoperative Valgus deformity in these studies broadly ranged from 3° to 15°. Robotic-assisted TKA consistently restored alignment toward the neutral mechanical axis. Alignment outcomes of valgus-focused studies are outlined in Table 5 comparing preoperative and postoperative HKA angles based on reported means or ranges. Furthermore, Marchand et al. reported correction within ± 3° of mechanical neutral in 96% of cases, while Gregori et al. demonstrated that 86.2% of knees achieved postoperative alignment within a defined safe zone (HKA 177–183°) [17, 18]. Wininger et al. also observed improved target al.ignment in robotically treated valgus knees compared to manual techniques (71.4% vs. 44.1%, *p* = 0.031) [32]. In terms of perioperative and functional outcomes, several studies emphasized improved soft tissue balancing and controlled deformity correction with robotic planning [17, 18, 31]. Chen et al. demonstrated that a sequential bone cutting technique could allow the avoidance of additional soft tissue release in a substantial proportion of knees while maintaining alignment within acceptable limits [24]. Gorur et al. demonstrated faster early PROM recovery and a shorter length of stay in robotic cohorts, with lower WOMAC scores (pain, stiffness, and function) at 1-year follow-up [31]. Gregori et al. reported significant improvements in KSS measures. Complication rates were comparably low across the studies [18].

**Table 5:** Preoperative vs. Postoperative HKA angles in Valgus Population undergoing Total Knee Arthroplasty

Study	Total Valgus Sample	*N* (Robotic)	Mean Pre-op HKA (°)	Mean Post-op HKA (°)	*N* (Convent.)	Mean Pre-op HKA (°)	Mean Post-op HKA (°)
Chapron 2025	74	74	182–192	181.0 ± 2.2 (AM approach)	N/A	N/A	N/A
Gorur 2024	74	44	NR	NR	30	NR	NR
Gregori 2025	58	58	≥ 183	181.1 (IQR 179–183, *p* < 0.0001)	N/A	N/A	N/A
Marchand 2021	152	152	NR	NR	N/A	N/A	N/A
Pacchiarotti 2025	43	23	173.97 ± 6.25	177.79 ± 2.13	20	179.19 ± 7.41	178.95 ± 2.39
Wininger 2023	62	28	−6.73 ± 2.16	0.24 ± 0.83	34	−8.58 ± 1.57	−0.60 ± 1.02

*Convent*. Conventional, *AM* Anteromedial approach, *HKA* Hip–knee–ankle angle, *IQR* Interquartile rang, *NR* Not reported, *N/A* Not applicable. Pre-op and post-op HKA values are mean ± SD unless otherwise specified

Overall, robotic-assisted TKA in valgus knees demonstrated consistent accuracy in coronal alignment, low outlier rates, and favourable early functional outcomes, with comparative data suggesting a potential benefit in cases of more severe deformity.

### Obese Patients

Two studies, including 469 knees (246 robotic-assisted, 223 conventional), evaluated robotic-assisted TKA in obese populations [33, 34]. In terms of radiographic outcomes, Richardson et al. reported improved postoperative coronal alignment in robotic cases among patients with BMI ≥ 35 kg/m^2^ [33]. As shown in Table 6 the Postoperative HKA deviation was significantly lower in robotic cases (2.0 ± 1.4° vs. 3.1 ± 3.2°, *p* = 0.040), with fewer radiographic outliers (> 3°) observed in the robotic group (15.0% vs. 30.5%, *p* = 0.043). Looking at perioperative outcomes, the operative time was shown to be longer in the robotic cohort. McCormick et al. assessed functional outcomes across BMI categories and found no significant interaction between obesity and surgical technique based on PROMs [34]. While non-obese patients showed higher SF-12 physical scores at two-year follow-up, robotic assistance did not provide any significant functional benefit with respect to BMI.

**Table 6:** Preoperative vs. Postoperative HKA angles in Obese Population undergoing Total Knee Arthroplasty

Study	Total Obese Sample	*N* (Robotic)	Mean Pre-op HKA (°)	Mean Post-op HKA (°)	*N* (Conventional)	Mean Pre-op HKA (°)	Mean Post-op HKA (°)
McCormick 2023	350	186	NR	NR	164	NR	NR
Richardson 2024	119	60	8.44 ± 4.86	1.97 ± 1.41	59	9.34 ± 5.26	3.11 ± 3.23

*HKA* Hip–knee–ankle angle, *NR* Not reported. Pre-op and post-op HKA values are reported as mean ± standard deviation

Overall, robotic-assisted TKA in obese patients was associated with improved radiographic alignment precision, although functional outcomes appeared similar between robotic and conventional techniques.

### Risk of Bias

Using ROBINS-I, 18 non-randomized studies were assessed, with most graded as moderate to serious risk of bias, primarily due to lack of control for confounding factors (e.g., age, BMI, comorbidities), selection bias inherent to cohort designs, and limited adjustment for baseline deformity severity (Supplementary Fig. 1). The single RCT (Tian et al.) was evaluated using the Cochrane Risk of Bias tool; it showed low risk in randomization and allocation concealment but high risk from lack of blinding and incomplete outcome data, resulting in an overall high risk of bias (Supplementary Fig. 2) [28]. Collectively, the evidence base remains predominantly retrospective and susceptible to confounding.

## Discussion

This study aimed to synthesize the available literature on the use of robot-assisted technology in complex primary and revision TKA. The most important finding of this review was that, across a range of complex indications, rTKA demonstrated consistent restoration of coronal alignment with perioperative and patient-reported outcomes comparable to cTKA. These findings suggest that robotic technology can achieve reliable alignment and clinical outcomes even in technically demanding knee arthroplasty cases.

Complex TKA presents several technical challenges, including severe coronal deformity, ligamentous instability, retained hardware, altered bony anatomy, and bone loss, all of which may complicate restoration of the mechanical axis and balanced soft tissues using conventional instrumentation [35]. These factors can reduce the utility and reliability of traditional intramedullary and extramedullary alignment guides and increase the likelihood of radiographic outliers following conventional total knee arthroplasty. Robotic-assisted systems have been posed as a potential solution to these challenges by allowing surgeons to perform detailed preoperative or intraoperative planning, execute precise bone resections, and dynamically assess soft-tissue balance throughout the arc of motion [36]. In the present review, alignment advantages were most apparent in cohorts with moderate-to-severe deformity, where robotic-assisted TKA consistently demonstrated fewer coronal alignment outliers and more reliable restoration of the mechanical axis compared with conventional techniques [26, 28].

Several studies also reported a reduced need for extensive soft tissue releases when robotic planning was used, suggesting that improved bone cut precision may facilitate deformity correction while preserving soft tissue balance [24, 26]. Collectively, these technical advantages may explain the consistent alignment accuracy observed across complex indications in the studies included in this review.

The findings of this review are consistent with previous literature demonstrating improved alignment precision with robotic-assisted TKA. In a case–control study evaluating conversion from UKA to TKA, Batailler et al. reported significantly fewer postoperative limb alignment outliers in the robotic group compared with conventional instrumentation [37]. Similarly, a meta-analysis by Onggo et al. demonstrated improved alignment accuracy across multiple axes and reduced intraoperative blood loss with robotic-assisted TKA compared with conventional techniques [36]. In the present review, robotic-assisted revision TKA demonstrated encouraging early outcomes, with postoperative alignment restored close to neutral and low re-revision rates across the included revision patients [19, 22]. Similar alignment advantages were observed in complex primary knees, particularly in patients with moderate-to-severe varus or valgus deformity, where robotic-assisted TKA produced fewer coronal alignment outliers compared with conventional techniques [17, 26, 28]. In obese populations, robotic assistance also improved radiographic alignment precision, although functional outcomes were largely comparable to conventional approaches [33, 34]. Collectively, these findings suggest that robotic systems may offer benefit in technically demanding knee arthroplasty cases where achieving accurate alignment and component positioning is more challenging. 

The included functional outcomes were generally positive. Patient-reported outcomes were measured using heterogeneous instruments, including KOOS Jr, OKS, and WOMAC, with variable follow-up intervals. Such heterogeneity is common in orthopedic literature, reflecting the lack of standardized reporting in knee arthroplasty research [38]. Despite this variability, most studies reported improvements in PROMs. Previous literature has established that relatively small gains can reflect meaningful improvements in pain, function, and quality of life, particularly in complex knee cases, underscoring the utility of these measures despite their heterogeneity [39, 40].

Several limitations should be considered when interpreting these findings. Most included studies were retrospective cohort designs (12/19), with only one randomized controlled trial, which increases the potential for selection bias and confounding. There was also heterogeneity in patient populations and definitions of complexity, including revision arthroplasty, severe deformity, and high BMI cohorts in varying proportions, limiting the potential for high-powered direct comparisons. Within studies, reporting of radiographic and patient-reported outcomes was inconsistent: changes in HKA angle were reported in 9/19 studies, MPTA in 4/19 studies, and clinical outcomes such as complication rate and length of stay were variably reported. Follow-up intervals also varied substantially across included studies, with several reporting only short-term outcomes (e.g., early functional scores, perioperative complications). This limits interpretation of functional recovery and implant-related outcomes, as early improvements may not reflect long-term performance or implant-survivorship. As part of the review process, title and abstract verification was supplemented by artificial intelligence-assisted screening using large language models (GPT-5.2 and Claude 4.5 Opus), which captured the majority of studies included by human reviewers, though a few studies were missed; this approach provided additional verification but highlighted the continued need for human oversight in complex systematic reviews. Finally, alignment strategies within each case were not known. Majority of cases targeted a neutral or mechanical axis given the complexity of deformity or revision setting. Eight different robotic platforms were used, ranging from imageless to image-based systems, each with different capabilities and learning curves, which may limit generalizability.

Future research should prioritize prospective, randomized controlled trials comparing robotic-assisted and cTKA in complex knee populations. Standardized reporting of alignment parameters (HKA, MPTA), patient-reported outcomes (WOMAC, KSS, OKS, KOOS Jr), and complications, along with longer-term follow-up, will be critical to determine whether improved alignment accuracy translates into better implant survivorship. While current data suggest favourable alignment and comparable clinical outcomes, future studies should also assess the cost-effectiveness and learning curve of robotic systems to evaluate whether the benefits outweigh the risks.

## Conclusion

Robotic-assisted knee arthroplasty appears to achieve consistent coronal alignment andoutcomes that are broadly comparable to conventional TKA in complex primary and revisioncases. Reported complication and re-revision rates were low and similar to those described inthe literature. However, given the predominance of retrospective studies, heterogeneousoutcome reporting, and limited long-term follow-up, these findings should be interpreted withcaution. Prospective studies with standardized outcomes, randomized study design andlongitudinal follow-up are needed to clarify whether improved alignment translates intosuperior long-term implant survivorship.

## Key references

- Marsh M, Newman S. Trends and developments in hip and knee arthroplasty technology. J Rehabil Assist Technol Eng. 2021.
  - Provides an overview of emerging technologies in arthroplasty, including robotic systems, and their potential to improve surgical precision and outcomes.
- Vivekanantha P, Son H, Bernardini L, Bouchard MD, Ayeni OR, Kay J. Evaluating the efficacy and efficiency of GPT-5 for automated title and abstract screening in orthopedic surgery systematic reviews. Curr Rev Musculoskelet Med. 2025.
  - Demonstrates the feasibility of using large language models to assist with title and abstract screening in orthopedic systematic reviews.
- Onggo JR, Onggo JD, De Steiger R, Hau R. Robotic-assisted total knee arthroplasty is comparable to conventional total knee arthroplasty: a meta-analysis and systematic review. Arch Orthop Trauma Surg. 2020; 140:1533–1549.
  - Shows that robotic-assisted TKA achieves comparable clinical outcomes to conventional techniques while improving implant positioning and alignment accuracy.

## Supplementary Information

Supplementary Material 1.

Supplementary Material 2.

Supplementary Material 3.

Supplementary Material 4.

## Data Availability

The data that support the findings of this study are derived from publicly available sources. Extracted data are included within the article and its supplementary materials.
