# Supplementary figures and images for: Robotic-Assisted Total Knee Arthroplasty in Complex Primary and Revision Cases: A Systematic Review
Source: Curr Rev Musculoskelet Med. 2026 Apr 9;19(1):32. doi: 10.1007/s12178-026-10026-x (PMC13065836; doi:10.1007/s12178-026-10026-x)

**Supplementary Figure 1:** Robins-I risk of bias for non-RCTs


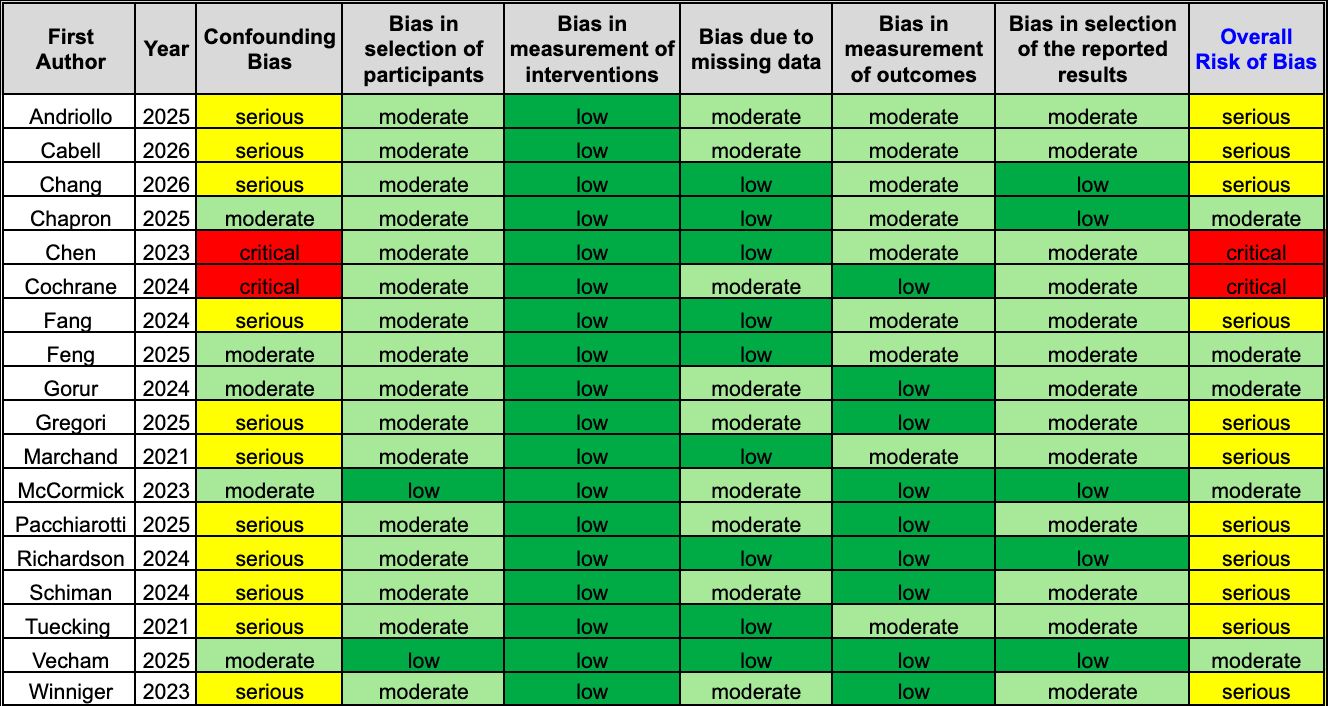

Supplement: Supplementary file 1 — Supplementary Material 1. [file 12178_2026_10026_MOESM1_ESM.docx]

**Supplementary Figure 2:** Cochrane Rob 2.0 for RCTs


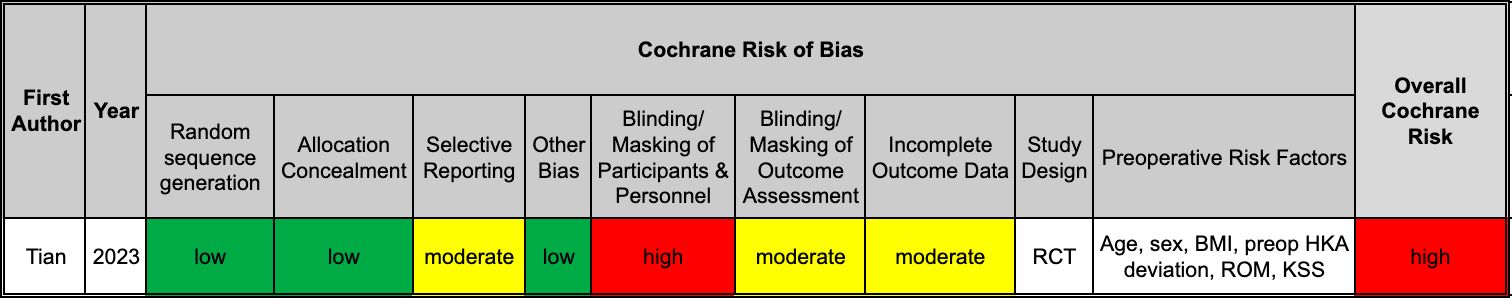

Supplement: Supplementary file 2 — Supplementary Material 2. [file 12178_2026_10026_MOESM2_ESM.docx]
