# Supplementary material for: Robotic-Assisted Total Knee Arthroplasty in Complex Primary and Revision Cases: A Systematic Review
Source: Curr Rev Musculoskelet Med. 2026 Apr 9;19(1):32. doi: 10.1007/s12178-026-10026-x (PMC13065836; doi:10.1007/s12178-026-10026-x)
Supplement: Supplementary file 3 — Supplementary Material 3. [file 12178_2026_10026_MOESM3_ESM.docx]

**Supplementary Table 1**: Search Strategy

| **Search strategy for MEDLINE, Cochrane Systematic Reviews, and EMBASE via OVID** |
| --- |
| 1. robotic.ti,ab |
| 2. robot-assisted.ti,ab |
| 3. MAKO.ti,ab |
| 4. ROSA.ti,ab |
| 5. VELYS.ti,ab |
| 6. NAVIO.ti,ab |
| 7. CORI.ti,ab |
| 8. total knee arthroplasty.ti,ab |
| 9. TKA.ti,ab |
| 10. complex.ti,ab |
| 11. severe deformity.ti,ab |
| 12. varus.ti,ab |
| 13. valgus.ti,ab |
| 14. obesity.ti,ab |
| 15. post-traumatic.ti,ab |
| 16. prior surgery.ti,ab |
| 17. osteotomy.ti,ab |
| 18. revision.ti,ab |
| 19. 1 OR 2 OR 3 OR 4 OR 5 OR 6 OR 7 |
| 20. 8 OR 9 |
| 21. 10 OR 11 OR 12 OR 13 OR 14 OR 15 OR 16 OR 17 OR 18 |
| 22. 19 AND 20 AND 21 |
