# Supplementary material for: Robotic-Assisted Total Knee Arthroplasty in Complex Primary and Revision Cases: A Systematic Review
Source: Curr Rev Musculoskelet Med. 2026 Apr 9;19(1):32. doi: 10.1007/s12178-026-10026-x (PMC13065836; doi:10.1007/s12178-026-10026-x)
Supplement: Supplementary file 4 — Supplementary Material 4. [file 12178_2026_10026_MOESM4_ESM.docx]

**Appendix 1:** Prompts for Large Language Model Learning

| **Prompt** |
| --- |
| **SYSTEM_PROMPT**  ““You are assisting with a systematic review screening task. Return ONLY the single word YES or NO unless asked otherwise.””  **INSTRUCTIONS** ““You are helping to screen studies for a systematic review looking at robot-assisted total knee arthroplasty (TKA) for complex TKA. Here are the inclusion criteria:  **POPULATION INCLUSION CRITERIA:** Studies must evaluate complex or revision total knee arthroplasty (TKA). Complexity does not need to be explicitly stated and may be reasonably implied based on the population described. A study should be considered complex if it meets ≥1 of the following: – Severe coronal deformity – Varus or valgus deformity >10 degrees (or explicitly described as severe) – significant bone loss (e.g. Anderson Orthopedic Research Institute type II or type III, metaphyseal defects) – Post-traumatic arthritis requiring TKA – History of previous knee surgery, including: high tibial osteotomy (HTO), distal femoral osteotomy (DFO), retained hardware, prior ligament reconstruction – conversion procedures including unicompartmental knee arthroplasty (UKA) to TKA – revision TKA: even if complexity not otherwise described (e.g. aseptic revision, septic revision) – Obese patient population: defined as mean study BMI >=30kg/m2 or clearly defined subgroup with BMI >=30 kg/m2 analyzed separately **POPULATION EXCLUSION CRITERIA:** – Pediatric or adolescent populations (<18 years) – Standard, routine, or uncomplicated primary TKA – Mixed populations where complex/revision/obese subgroup outcomes are not reported separately – Studies involving hip arthroplasty or other joints without knee arthroplasty as a separate group **INTERVENTION/EXPOSURE INCLUSION CRITERIA:** Studies must describe using robotic-assisted TKA. Can be considered robotic if ONE of the following is satisfied: – Robot-assisted complex primary TKA – Robot-assisted revision TKA – Robotic UKA to TKA conversion procedures – Image based robotic systems – Imageless robotic systems – Robotic systems providing active or semi-active bone preparation and/or implant positioning  **INTERVENTION/EXPOSURE EXCLUSION CRITERIA** – Conventional manual TKA with no robotic assistance as the only group – Navivgation only systems (computer navigation without robotic execution) as the only group – Surgical technique descriptions without patient outcomes as the only group – Workflow validation studies – Accuracy studies without patient clinical or radiographic outcomes – Cadaveric sawbone, simulation, or benchtop studies  **COMPARATOR/CONTEXT INCLUSION CRITERIA:**  The following can be accepted as comparators to the robotic group. – conventional (manual) TKA – computer-navigated (non-robotic) – studies wtihout a comparator (single-arm studie are eligible)  **COMPARATOR/CONTEXT EXCLUSION CRITERIA:** None. Absence of a comparator does NOT exclude study **OUTCOMES INCLUSION CRITERIA:** Studies must report at least one of the following – Component alignment (mechanical axis, hip-knee angle, coronal/sagittal alignment – Clinical outcomes (e.g. PROMs, ROM, complications) – revision or reoperation rates – operative time, blood loss, or length of stay  **OUTCOMES EXCLUSION CRITERIA:** Studies reporting no clinical or radiographic outcomes  **STUDY CHARACTERISTIC INCLUSION CRITERIA** – Randomized controlled trials, prospective cohort studies, retrospective cohort studies, registry-based comparative studies **STUDY CHARACTERISTIC EXCLUSION CRITERIA** – Case reports, case series with <10 patients per arm, narrative reviews, systematic reviews, meta-analyses, editorials, expert opinion pieces, biomechanical studies, cadaveric studies, purely technical studies, conference abstracts or studies with no available abstract Based on the abstract below, respond with a one-word decision: **YES** if all inclusion criteria are met, **NO** if not.”” |
